# Vitamin D status in Psoriasis: impact and clinical correlations

**DOI:** 10.1186/s40795-022-00610-y

**Published:** 2022-10-19

**Authors:** Ghulam Hassan Bhat, Sadaf Guldin, Mosin Saleem Khan, Mir Yasir, Ganesh Prasad

**Affiliations:** 1 Department of Biochemistry, Yenepoya Medical College Hospital, 575018 Mangalore, India; 2 Department of Microbiology, Yenepoya Medical College Hospital, 575018 Mangalore, India; 3 Department of Biochemistry, Government Medical College Baramulla and Associated Hospitals, Kanth Bagh, 193101 Baramulla, J&K India; 4 Department of Plastic and Reconstructive Surgery, Sher-I-Kashmir Institute of Medical Sciences (SKIMS), Soura, 190011 Srinagar, J&K India

**Keywords:** Vitamin D, 25-hydroxy vitamin D, Psoriasis, Severity, Autoimmunity

## Abstract

Psoriasis is a continuing, periodic, immune‑mediated, fiery skin disease branded by hyper proliferation of epidermal keratinocytes and accompanying with inflammatory cellular infiltrate in both dermis and epidermis. Immunomodulation could be an important effect of vitamin D in Psoriasis. This case-control study was designed to measure serum 25-hydroxy vitamin D levels in patients with psoriasis and healthy controls and to find out clinical correlation, if any. Six hundred two (n = 602) subjects (285 cases and 317 controls) were taken for the study. Cases and controls were frequency matched with respect to age and gender. Various demographic and clinical details were taken using a questionnaire. Chemiluminescence Micro Particle Immunoassay was used to estimate serum 25-hydroxy vitamin D levels. The vitamin D deficiency in psoriasis patients was 60.0% vs. 17.5% in controls (P < 0.001) with mean vitamin D levels of 28.3 ± 13.9 ng/ml in psoriasis patient’s vs. 37.9 ± 9.7 ng/ml in controls. Vitamin D deficiency was found to be associated with psoriasis independently of gender, age, smoking status, family history, hypertension, chronic medication, nail changes, duration of symptoms and severity of disease. Vitamin D levels were seven times lower in patients with Psoriasis as compared to controls. Reduced vitamin D levels are related to duration and clinical severity of the disease. Early detection of vitamin D deficiency and timely intervention could lead to better clinical outcome and improved quality of life in psoriasis patients.

## Introduction

Psoriasis is a chronic, disfiguring, proliferative and inflammatory ailment of skin characterized by keratinocyte hyper-proliferation, abnormal keratinocyte differentiation and immune-cell infiltration into the epidermis and dermis [1]. The most typical abrasion involves red, crusty, tightly demarcated, indurated plaques, existing mainly over scalp and extensor surfaces. The ailment is extremely inconstant in duration and spell of flares [2]. Disease presents with a bimodal distribution of age at onset, with a peak between 15 and 20 years and another peak between 55 and 60 years [3]. Psoriasis is associated with Psoriatic Paronychia and Psoriatic Arthritis [3]. There are several psoriasis phenotypes. The most common clinical variant effecting nearly 85–90% of all patients is *Psoriasis Vulgaris*, recognized by elevated, distinct, erythematous lesions with silvery scale, mostly effecting scalp, knees, sacral region and elbows. Overall prevalence of Psoriasis ranges from 0·1% in east Asia to 1·5% in western Europe; highest being in high-income countries. Prevalence and incidence is lower in children compared to adults and equal across both genders [4].

Vitamin D is a fat soluble steroid hormone. 25-hyroxy vitamin D is the most stable form with a half-life of 2–3 weeks, hence reliable medical indicator of vitamin D status [5]. Vitamin D has pleotropic functions. It controls calcium homeostasis by acting as a hormone as well as exercises autocrine/paracrine effects on CYP27B1 and VDR expressing tissues. In addition, vitamin D may reduce the risk of psoriasis by inhibiting T-cell proliferation/Th1 development, tempering antigen presenting cell (APCs) function, bringing hypo-responsiveness to antigens, decreasing the levels of IL-2, IL-17, IL-8, INF-α and INF-γ, enhancing manufacture of IL-10 and regulatory T cells [5, 6]. Vitamin D regulates the synthesis and release of human beta-defensin 2 (HBD2), antimicrobial peptides and cathelicidin which take part in the etiopathogenesis of psoriasis [5, 6]. Various studies have reported the involvement of vitamin D in the pathogenesis of different skin diseases, including Psoriasis [7, 8].

In this context, due to uncertain mechanisms, the intricate relationship between vitamin D levels and chronic autoimmune or inflammatory diseases such as Psoriasis, becomes obvious. So, this case-control study was designed to determine the serum 25-hydroxy vitamin D levels and its association, if any, with various socio-demographic and clinicopathological parameters of patients with psoriasis.

## Materials and methods

### Study design

This was a case-control study conducted by the Departments of Biochemistry in collaboration with the Department of Dermatology, Venerology and Leprosy. A written informed consent was obtained from study subjects. The study was approved by University Ethics Committee in accordance with the ethical standards of the World Medical Association Declaration of Helsinki involving human subjects and/or animals.

### Study subjects and sample size

A total of two hundred eighty-five (n = 285) patients with psoriasis attending Department of Dermatology, Venerology and Leprosy were enrolled for the present study over a period of two years. Three hundred seventeen (n = 317) controls were randomly selected from a pool of healthy volunteers who visited the hospital for health check-up during the same period. Cases and controls were frequency matched with respect to age and gender. Keeping power of study as 80% (0.8), sample size was calculated using statistical software G POWER 20.0.

### Inclusion criteria

All the psoriasis patients confirmed by PASI score and healthy controls with > 18 years of age were included in the study. In order to neglect the effect of diet or outdoor habits on serum vitamin D levels, all the cases as well as controls included in the study received at least 20–30 min of sunlight on the face and forearms during midday, 2–3 times a week in addition to diet adequate in vitamin D. Patients on topical therapy were allowed to participate after 1 month of discontinuation of the therapy.

### Exclusion criteria

As liver disease, kidney disease, autoimmune diseases, inflammatory bowel disease, malabsorption syndromes, malignancy, pregnancy or any acute febrile illness have an effect on total vitamin D levels, the cases as well as controls having those conditions were excluded from the study. Patients on vitamin D supplements and systemic therapy like methotrexate, cyclosporine, were not allowed to participate. In addition, psoriasis patients on biologics were excluded from the study.

### Diagnostic criteria and severity score of Psoriasis

A detailed clinical examination including general, systemic and dermatological examination was carried out. Disease severity was assessed as per *Psoriasis Area Severity Index (PASI)* and *body surface area (BSA)* participation [9]. PASI is a useful tool in monitoring the response of psoriasis to any therapeutic regimen. The PASI pools severity of lesions and the area affected, into a solitary score between the range of 0 to 72. Four sites of affection, viz. Head (h), Upper limb (u), Trunk (t), and Lower limbs (l) are separately scored. Morphologic scoring of psoriasis plaques is done by evaluation of three parameters viz. Erythema [E], Induration [I] and Desquamation [D], each of which have a severity scale of 0 to 4 (0: nil, 1: mild, 2: moderate, 3: severe, and 4: very severe). Then the area-wise percentage involvement is multiplied by prior calculated scores for each site (1: less than 10% area, 2: 10–29%, 3: 30–49%, 4: 50–69%, 5: 70–89% and 6: 90% or more are involved in psoriasis). Since head, upper limbs, trunk, and lower limbs denote 10%, 20%, 30% and 40% of the body surface area separately, they are given matching weightage in scoring by multiplying their scores by 0.1, 0.2, 0.3, 0.4 respectively. Hence the final formula for calculating PASI;

$$\begin{array}{c}PASI = 0.1(Eh + Ih + Dh)Ah + 0.2(Eu + Iu + Du)Au\\+ 0.3(Et + It + Dt)At + 0.4(El + Il + Dl)Al\end{array}$$

The PASI is the utmost authenticated unbiased scheme to quantify the severity of psoriasis [10, 11]. The PASI score is delicate and mirrors improvement or worsening of disease [12]. PASI Score can vary between 0 and 72 in steps of 0.1(10) [13]. On the basis of PASI score psoriasis patients are classified as mild, moderate and severe [14]. PASI score of < 3 was indicator of mild disease, 3–10 as moderate disease and > 10 as severe psoriasis [14].

### Sample collection

03 ml of venous blood sample was drawn from each psoriasis patient and control subject in Red top or clot activators vials, thereafter, centrifuged at 3000–4000 rpm for separation of serum. Serum was stored at -20 °C till further analysis.

### Estimation of serum 25-hydroxy vitamin D levels

The 25-hydroxy vitamin D test was performed using *VITROS 5600 Integrated System* (Ortho Clinical Diagnostics, Canada). It includes a Chemiluminescence immunoassay technique which uses low pH denaturant for the release of binding protein from serum 25-hydroxy vitamin D and the later competition of free 25-hydroxy vitamin D with horseradish peroxidase (HRP) tagged reagent against monoclonal anti-vitamin D adhered to the wells. Chemiluminescent reaction measures the bound HRP conjugate [15]. A reagent containing luminogenic substrates and an electron transfer agent, is added to the wells. The HRP in the bound conjugate catalyses the oxidation of the luminol derivative, which has been previously added to wells, producing light which is increased and prolonged due to prior addition of electron transfer agent. The amount light emitted (HRP conjugate bound) is indirectly proportional to the concentration of 25-hydroxy vitamin D present in serum sample. The incubation time for the reaction is about 16 min at 37 °C and a reaction sample volume of 60 µl is needed. Serum 25-hydroxy vitamin D level of < 20 ng/ml was considered as “Deficient”; 20-29.99 ng/ml was as “Insufficient”; 30–100 ng/ml as “Sufficient” and > 100 ng/ml as “Potentially toxic” [16].

### Statistical analysis

Statistical analysis was done by SPSS 23.0 statistical package (SPSS Inc., Chicago IL, USA). The cases and controls were in Hardy-Weinberg equilibrium. The age and 25-hydroxy vitamin D levels were analysed by student’s t-test between the groups. Univariate and multivariate logistic regression analysis was done to compare 25-hydroxy vitamin D as categorical variable between cases and controls. χ^2^-test was used to compare the variables such as age, sex, smoking status, etc. between the cases and controls with respect to 25-hydroxy vitamin D as categorical variable. The association of 25-hydroxy vitamin D levels with the risk of psoriasis was estimated by odds ratios (OR) with 95% confidence intervals (95% CI). P value of ≤ 0.05 was considered as significant.

## Results

### Characteristics of cases and controls

A total of 285 Psoriasis patients were taken for the study. These included 61.8% (176 of 285) males and 38.2% (109 of 285) females. 95 out of 285 (33.3%) patients were < 40 years of age while as 190 of 285 (66.7%) were ≥ 40 years of age. The mean age of patients was 44.6 ± 13.5 years ranging from 20 to 70 years. 73.0% (208 of 285) of patents were non-smokers while as 27.0% (77 of 285) were smokers. Almost 11.6% (33 of 285) patients had family history of Psoriasis while as 31.9% (91 of 285) had alcohol abuse. 177 of 285 (62.1%) patients had symptoms for the past < 5 years while as 108 of 285 (37.9%) had symptoms for the past ≥ 5 years. The maximum duration of disease/symptoms was 20 years and the minimum duration was 3 months with a mean duration of 4.32 years. Among psoriasis patients 4.2% (12 of 285) had mild, 43.9% (125 of 285) has moderate and 51.9% (148 of 285) has severe disease.

Apart from cases, a total of 317 controls were also included for the study. The cases and controls were frequency matched with respect to age and gender. Almost 60.0% (189 of 317) of controls were males and 40.0% (128 of 317) were females. 112 out of 317 (35.3%) controls were < 40 years of age while as 205 of 317 (64.7%) were ≥ 40 years of age. The mean age of controls in years was 43.6 ± 13.7 within a range of 20 to 69 years. 80.1% (254 of 317) of controls were non-smokers while as 19.9% (63 of 317) were smokers. Table 1 contains demographic and clinicopathological characteristics of psoriasis cases and controls included in the study.

**Table 1:** Socio-Demographic and Clinicopathological parameters of Psoriasis cases and healthy controls

Parameters	Controls n = 317 (%)	Cases n = 285 (%)	*P* value
**Gender** Male Female	189 (59.6) 128 (40.4)	176 (61.8) 109 (38.2)	0.6
**Age in years**	43.6 ± 13.7	44.6 ± 13.5	0.5
**Age group** < 40 years ≥ 40 years	112 (35.3) 205 (64.7)	95 (33.3) 190 (66.7)	0.6
**Smoking status** Non-Smoker Smoker	254 (80.1) 63 (19.9)	208 (73.0) 77 (27.0)	**0.04**
**Family History** No Yes	-	252 (88.4) 33 (11.6)	**-**
**Alcoholism** No Yes	-	194 (68.1) 91 (31.9)	-
**Hypertension** No Yes	-	146 (51.2) 139 (48.8)	**-**
**Chronic Medication** No Yes	-	223 (78.2) 62 (21.8)	-
**Nail Changes** No Yes	-	121(42.5) 164 (57.5)	**-**
**Duration of Symptoms** < 5 years ≥ 5 years	-	177 (62.1) 108 (37.9)	-
**Severity of Disease** Mild Moderate Severe	-	12 (4.2) 125 (43.9) 148 (51.9)	**-**

### 25-hydroxy vitamin D levels in cases vs. controls

On comparison of serum 25-hydroxy vitamin D levels between cases and controls, 40.7% (116 of 285), 28.4% (81 of 285) and 30.9% (88 of 285) psoriasis patients had *sufficient (S), insufficient (I)* and *deficient (D)* 25-hydroxy vitamin D levels respectively, compared to 82.0% (260 of 317) and 18.0% (57 of 317) of healthy controls having *sufficient (S)* and *insufficient (I)* 25-hydroxy vitamin D levels respectively. Healthy controls were not having *deficient (D)* 25-hydroxy vitamin D levels. There was a statistically significant difference in 25-Hydroxy vitamin D levels of cases and controls (P < 0.001). On multivariate analysis, 25-hydroxy Vitamin D levels were significantly decreased in psoriasis cases compared to controls when adjusted for age, gender, and smoking status (P < 0.001). Table 2 depicts the association between various groups of cases and controls with respect to 25-hydroxy vitamin D levels.

**Table 2:** Association of vitamin D levels between Psoriasis cases and healthy controls

Vitamin D levels	Controls n = 317 (%)	Cases n = 285 (%)	OR (95% CI)	*P* value	*OR (95% CI)	*P value
Sufficient Insufficient Deficient	260 (82.0) 57 (18.0) 00 (0.0)	116 (40.7) 81 (28.4) 88 (30.9)	1.00 (ref.) 3.1 (2.1–4.8) 198.5 (27.3-1444.2)	**< 0.0001** **< 0.0001**	1.00 (ref.) 7.2 (4.4–11.9) 201.2 (29.6-1487.6)	**< 0.0001** **< 0.0001**

^*^ *Adjusted with respect to gender, age and smoking status*

The mean 25-hydroxy vitamin D levels of cases in ng/ml was 28.3 ± 15.0 within a range of 8.0 to 89.2 compared to 37.8 ± 9.8 in controls within a range of 20.5–62.0. A statistical significance was prominent between 25-hydroxy vitamin D levels of cases and controls (P < 0.0001). Table 3 contains levels of 25-hydroxy vitamin D in cases and controls in terms of mean ± SD.

**Table 3:** Levels of vitamin D in Psoriasis cases and healthy controls

Parameters	Controls Mean ± SD (Range)	Cases Mean ± SD (Range)	P value
**Vitamin D levels in ng/ml**	37.8 **±** 9.8 (20.5–62.0)	28.3 **±** 15.0 (8.0-89.2)	**< 0.0001**

### Stratification analysis with respect to 25-hydroxy vitamin D levels

Table 4 depicts the association of serum 25-hydroxy vitamin D levels with socio-demographic and clinicopathological parameters of psoriasis cases and healthy controls. Due to the increased risk of psoriasis with 25-hydroxy vitamin D insufficiency and deficiency, individuals with *Insufficient (I)* and *Deficient (D)* 25-hydroxy vitamin D levels were clubbed and compared with individuals having *Sufficient (S)* vitamin D levels. In addition, as the frequency of individuals having *Insufficient (I)* and *Deficient (D)* 25-hydroxy vitamin D levels was less, they were clubbed together for statistical viability (Table 4).

**Table 4:** Association of serum 25-hydroxy vitamin D levels with socio-demographic and clinicopathological parameters of Psoriasis cases and healthy controls

Socio-Demographic/ Clinicopathological parameters	25-hydroxy vitamin D levels in Controls			25-hydroxy vitamin D levels in Psoriasis Cases			OR (95%CI)	P value
	**n = 317 (%)**	**S** **260 (82.0)**	**I + D** **57 (18.0)**	**n = 285 (%)**	**S** **116 (40.7)**	**I + D** **169 (59.3)**	**6.6 (4.5–9.6)**	**< 0.0001**
**Gender** Male Female	189 (59.6) 128 (40.4)	163 (86.2) 97 (75.8)	26 (13.8) 13 (24.2)	176 (61.8) 109 (38.2)	103 (58.5) 13 (11.9)	73 (41.5) 96 (88.1)	4.4 (2.6–7.4) 23.1 (11.4–46.8)	< 0.0001 < 0.0001
**Age group** < 40 years ≥ 40 years	112 (35.3) 205 (64.7)	96 (85.7) 164 (80.0)	16 (14.3) 41 (20.0)	95 (33.3) 190 (66.7)	49 (51.6) 67 (35.3)	46 (48.4) 123 (64.7)	5.6 (2.9–10.9) 7.3 (4.6–11.5)	< 0.0001 < 0.0001
**Smoking status** Non-Smoker Smoker	254 (80.1) 63 (19.9)	197 (77.6) 63 (100.0)	57 (22.4) 00 (0.0)	208 (73.0) 77 (27.0)	72 (34.6) 44 (57.1)	136 (65.4) 33 (42.9)	6.5 (4.3–9.8) 48.3 (6.3-366.2)	< 0.0001 < 0.0001
**Family History** No Yes	-	-	-	252 (88.4) 33 (11.6)	88 (34.9) 28 (84.8)	164 (65.1) 05 (15.2)	1.00 (Ref.) 0.09 (0.03–0.26)	**< 0.0001**
**Alcoholism** No Yes	-	-	-	194 (68.1) 91 (31.9)	87 (44.8) 29 (31.9)	107 (55.2) 62 (68.1)	1.00 (Ref.) 1.7 (1.1–2.9)	**0.04**
**Hypertension** No Yes	-	-	-	146 (51.2) 139 (48.8)	76 (52.1) 40 (28.8)	70 (47.9) 99 (71.2)	1.00 (Ref.) 2.6 (1.6–4.3)	**< 0.0001**
**Chronic Medication** No Yes	-	-	-	223 (78.2) 62 (21.8)	108 (48.4) 08 (12.9)	115 (51.6) 54 (87.1)	1.00 (Ref.) 6.3 (2.8–13.9)	**< 0.0001**
**Nail Changes** No Yes	-	-	-	121(42.5) 164 (57.5)	69 (57.0) 47 (28.7)	52 (43.0) 117 (71.3)	1.00 (Ref.) 3.3 (2.0-5.4)	**< 0.0001**
**Duration of Symptoms** < 5 years ≥ 5 years	-	-	-	177 (62.1) 108 (37.9)	87 (49.2) 29 (26.9)	90 (50.8) 79 (73.1)	1.00 (Ref.) 2.6 (1.6–4.4)	**0.04**
**Severity of Disease** Mild Moderate Severe	-	-	-	12 (4.2) 125 (43.9) 148 (51.9)	05 (41.7) 96 (76.8) 15 (10.1)	07 (58.3) 29 (23.2) 133 (89.9)	1.00 (Ref.) 0.2 (0.06–0.7) 6.3 (1.8–22.4)	**0.02** **0.004**

* S = Sufficient, I = Insufficient, D = Deficient*

The frequency of males as well as females having *Insufficient (I)* and *Deficient (D)* 25-hydroxy vitamin D levels (I + D) was significantly higher in psoriasis patients compared to controls (P < 0.0001), although the difference in 25-hydroxy vitamin D levels between female cases and controls was always greater when compared to male cases and controls (OR; 23.1 vs. 4.4). Furthermore, our study found a significant association of low 25-hydroxy vitamin D levels (I + D) with the risk of developing psoriasis irrespective of age and smoking status (P < 0.0001), although in smokers the risk of developing psoriasis was many fold higher due to low 25-hydroxy vitamin D levels (I + D) compared to non-smokers (OR: 48.3 vs. 6.5). Interestingly, our observation revealed a significantly high number of psoriasis cases having low 25-hydroxy vitamin D levels (I + D) but without family history of psoriasis (OR = 0.09; P < 0.0001). Low 25-hydroxy vitamin D levels were significantly present in psoriasis patients with alcoholic abuse (P = 0.04), hypertension (P < 0.0001), chronic medication (P < 0.0001), nail changes (P < 0.0001) and duration of symptoms for ≥ 5 years (P = 0.04).

On taking severity of disease into consideration, there was a significant difference between the 25-hydroxy vitamin D levels of psoriasis patients with mild and moderate disease (P = 0.02). Almost 90% of psoriasis patients with severe disease had low 25-hydroxy vitamin D levels as compared to only 58.3% of patients with mild disease having low 25-hydroxy vitamin D levels (OR = 6.3; P = 0.004).

## Discussion

The prevalence of psoriasis is 0·1% in east Asia to 1·5% in western Europe [4]. The disease may be triggered by a combination of genetic factors, environmental agents in addition to random factors [17]. Although, the prevalence of psoriasis is considered to be balanced between the sexes [18], our study noted higher prevalence of psoriasis in males. Our observation correlates well with the results of Metha et al. who found male: female ratio of 4:1 in psoriasis patients [19]. With respect to age, psoriasis shows a bimodal distribution with 70% of cases showing a peak onset between 15 and 20 years of age and 30% of cases showing a peak onset between 55 and 60 years [4]. In our study the maximum number of patients belonged to age group ≥ 40 years with mean age of 44.6 ± 13.5 years at onset. Age at disease onset, based on a cut-off of 40 years, is an acknowledged discriminator, bifurcating psoriasis into early and late-onset disease [20]. Opposed to our finding, onset at ≤ 40 years has been reported in approximately 75.0% of psoriasis patients globally [20]. In our study approximately 20.0% of the patients had a history of smoking. As compared to general population, patients with psoriasis are more than twice as likely to smoke cigarettes [21]. Furthermore, severity of psoriasis has been attributed to heavy tobacco intake [22]. Surprisingly, occasional reports have speculated the negative effect of smoking on psoriasis [23]. In our study 12.5% of psoriasis patients were having family history of the disease which is in agreement with the previous literature [24]. The genetic load of psoriasis has been well described in the literature, as has the effect of family history of psoriasis on disease outcomes [24, 25]. A large number of genetic loci have been described in psoriasis in the last decade by the genome-wide association studies. Fewer studies conducted to identify psoriasis risk variants showed the significant differences in the genetic architecture of psoriasis may also be reflected in the phenotypic characteristics of these diseases [26]. The simple difference in *psoriasis* versus *psoriasis with family history* may point to a deeper genetic difference in familial cases of psoriatic disease and may be an important factor to consider in the era of personalized medicine. In our study, 30% of the patients were alcoholics. Most of the studies have reported a positive correlation between alcohol intake and the extent of psoriasis [27]. Alcohol may affect psoriasis through several mechanisms, such as increased susceptibility to infections, stimulation of lymphocyte and keratinocyte proliferation, and production of pro-inflammatory cytokines [28]. However as per few previous studies no association of psoriasis with alcohol consumption was observed [29]. In our study, almost half of the enrolled psoriasis patients were hypertensive. As per previous studies, an increased frequency of elevated blood pressure in severe psoriasis patients was detected compared to milder cases [30]. Although, a specific pathophysiological mechanism linking hypertension with psoriasis has not been determined, but increased blood pressure in psoriasis patients can be related to an increased level of angiotensin-converting enzyme, endothelin-1 (ET-1) and rennin [31]. In our study 20.0% of the cases were on chronic medication. Psoriasis can be triggered or aggravated by various environmental factors, particularly infections and drugs. *Streptococcal* infection is strongly associated with *Guttate Psoriasis.* As per Mallbris et al. 63.0% of patients with guttate phenotype had acute streptococcal pharyngitis at disease onset [32]. Initiation or worsening of the disease has been linked to the use of various drugs such as angiotensin-converting enzyme, antimalarial agent inhibitors, lithium, β-blockers and IFN-α [33]. Nail changes were observed in 52.5% of enrolled patients. Previous studies have established that nail changes are involved in 10.0 − 55.0% of patients suffering from psoriasis [34].

The relation between 25-hydroxy vitamin D and psoriasis has been studied since the 1930s. Out of serendipity, Morimoto et al. in 1985 observed that the administration of 25-Hydroxy vitamin D could improve psoriasis in some cases [35]. The immune modulatory role of 25-hydroxy vitamin D has been well established, with its deficiency leading to Psoriasis [36]. In our study, the serum 25-hydroxy vitamin D levels were significantly lower in psoriasis patients compared to healthy controls, and the lowest measure was obtained from patients with severe psoriasis *(*Tables 2 and 3*).* Our observation was in consistency with the studies of Ricceri et al. and Orgaz-Molina et al. along with majority of other studies, wherein they showed low levels of serum 25-hydroxy vitamin D, in patients with psoriasis than in control subjects [37–40]. Contrary to our observations, some of the studies did not find any association between 25-hydroxy vitamin D levels and risk of psoriasis [41]. Gisondi et al. found that the prevalence of 25-hydroxy vitamin D deficiency (< 20 ng/ml) was 57.8% in subjects with psoriasis and 29.7% in healthy controls (P < 0.001) [42].

We observed that serum 25-Hydroxy vitamin D levels of female psoriasis cases were significantly high compared to controls of same gender (OR = 23.1; P < 0.0001). As per previous studies, the 25-hydroxy vitamin D levels were reduced in females leading to various immune and cardiovascular disorders [43, 44]. This link may be due to some modifiable factors like wearing cosmetics with sunscreen effect, lesser sun exposure time or too much clothing in female folk, especially due to religious bindings in Kashmir valley. In contradiction with our study, the studies conducted by Ricceri et al. and Orgaz-Molina et al. did not find significant differences between gender and 25-hydroxy vitamin D levels [37, 38]. Although it has been speculated that 25-hydroxy vitamin D deficiency in elderly could be due to age related decrease in the 25-hydroxy vitamin D producing capacity of skin; from lack of exposure to sunlight or from a deficient dietary intake [45] but our study reported a marked decrease in 25-hydroxy vitamin D levels in psoriasis cases compared to controls in both age groups (< 40 and ≥ 40 years). Moreover, as per a Turkish study the highest prevalence of 25-Hydroxy vitamin D deficiency was found in the age group 20–30 years [46]. As per our data, smokers as well as non-smokers with 25-hydroxy vitamin D deficiency were at a higher risk of developing psoriasis. A negative effect of smoking on serum 25-hydroxy vitamin D levels was found in a Swedish case-control study [47]. Similarly, slightly reduced concentrations of 25-hydroxy vitamin D has been described in healthy American postmenopausal female smokers [48]. Several hypotheses have been put forward concerning the mechanisms by which smoking affects 25-hydroxy vitamin D levels, the main focus being on the antiestrogenic effect [49]. As per our study, most of the psoriasis patients with decreased 25-hydroxy vitamin D levels were hypertensive. Metabolic syndrome, especially hypertension is a common link between 25-hydroxy vitamin D deficiency and risk of psoriasis [50]. However, one of the case-control studies did not observe any correlation of serum 25-hydroxy vitamin D levels with blood pressure, HDL cholesterol, triglycerides or blood sugar levels [51]. 25-hydroxy vitamin D and Calcium have role in proper growth and differentiation of nail unit [52] and we observed a very high frequency of nail changes in psoriasis patients with reduced 25-hydroxy vitamin D levels which is in line with several studies who have reported reduced levels of 25-hydroxy vitamin D and calcium in psoriasis [53]. As per our study, duration of symptoms was longer in psoriatic patients with reduced 25-hydroxy vitamin D levels. It is a known statement that psoriasis patients, except those undergoing phototherapy, have a tendency to keep their affected skin covered over the years which leads to reduced UV exposure resulting in decreased 25-hydroxy vitamin D levels. Therefore, chronic psoriasis patients could probably have reduced 25-hydroxy vitamin D levels [54, 55]. In the current study, there was a significant decrease in the levels of serum 25-hydroxy vitamin D in severe psoriasis patient group compared with mild disease group which is in agreement with the study by Abdalla and Abdrabo [56]. Mattozzi et al. significantly related severity of disease with reduced serum 25-hydroxy vitamin D levels [57]. In fact, the high prevalence of 25-hydroxy vitamin D insufficiency is defined as a global health problem, and most experts recommend a level ≥ 30 ng/ml as sufficient [58].

It has been established that low concentration of 25-hydroxy vitamin D promotes keratinocyte proliferation and maturation while at higher concentration has an inhibitory effect [59, 60]. 25-hydroxy vitamin D downregulates the expression and production of several pro-inflammatory cytokines including TNF-α, IL-1β, IL-6, and IL-8 [61], thereby establishing its anti-inflammatory effect on the inflammatory profile of psoriasis [62, 63]. As per previous studies, oral vitamin D supplementation could decrease psoriasis-related comorbidity [64], and could be used as a treatment option in psoriatic patients [65, 66]. On the other hand, some studies found no significant association between vitamin D supplementation and risk of psoriasis [67]. Although the potential use of oral vitamin D supplementations to treat vitamin D deficient psoriasis patients has been strongly supported by a comprehensive meta-analysis including the results of clinical trials, 25-hydroxy Vitamin D supplements didn’t improve the symptoms in psoriatic patients with normal serum 25-hydroxy vitamin D levels [68].

## Conclusion

Our study and the current available data show that 25-hydroxy vitamin D deficiency is common in severe form of psoriasis. Maintenance of serum 25-hydroxy vitamin D levels above 30 ng/ml could contribute to a better evolution when it comes to autoimmune and inflammatory diseases, such as psoriasis. Large randomized controlled trials in the said population be accomplished to see whether increase in 25-hydroxy vitamin D levels would result in a statistically significant clinical improvement.

## Data Availability

The datasets generated and/or analysed during the current study are not publicly available due strict institutional policy of data encryption but are available from the corresponding author on reasonable request.
